# Renal damage induced by pemetrexed causing drug discontinuation: a case report and review of the literature

**DOI:** 10.1186/s13256-017-1348-6

**Published:** 2017-07-28

**Authors:** Yassir Sbitti, Hafsa Chahdi, Khaoula Slimani, Adil Debbagh, Anouar Mokhlis, Abderrahmane Albouzidi, Fahd Bennani, Hassan Errihani, Mohamed Ichou

**Affiliations:** 1 Department of Medical Oncology, University Military Hospital, Rabat, 10000 Morocco; 2 Department of Pathology, University Military Hospital, Rabat, 10000 Morocco; 3 Department of Medical Oncology, National Institute of Oncology, Rabat, 10000 Morocco

**Keywords:** Pemetrexed, Renal damage, Non-small cell lung carcinoma

## Abstract

**Background:**

Pemetrexed maintenance therapy holds tremendous potential in improving the survival of patients with advanced pulmonary adenocarcinoma. Major side effects include myelosuppression and cutaneous reactions. However, little data are available on pemetrexed nephrotoxicity. Our case describes clinically relevant renal events leading to treatment discontinuation in routine practice.

**Case presentation:**

We report a case of a 69-year-old Moroccan man treated for metastatic non-small cell lung cancer. He was not on any other medications and he did not receive any nephrotoxic agents. He was exposed to intravenously administered contrast from thoracoabdominal computed tomography in the week of his last pemetrexed treatment. He developed kidney disease related to pemetrexed. He was submitted to renal biopsy, which showed acute tubular damage and interstitial fibrosis. His kidney function remained impaired, but stable, after discontinuation of pemetrexed therapy. He died 5 months later.

**Conclusions:**

Medical oncologists should be aware of renal adverse events for patients with advanced non-small cell lung cancer eligible for pemetrexed maintenance therapy. Suggestions for mitigating the risk for renal toxicities (dehydration, non-steroidal anti-inflammatory drugs and zoledronic acid, radiocontrast agents) during pemetrexed maintenance should be followed to enable early detection and management of this adverse event.

## Background

Maintenance therapy has emerged as a novel therapy for patients with non-progressive advanced non-squamous cell lung cancer after induction. The value of maintenance therapy is now statistically established [1, 2]. However, not all patients need maintenance therapy and the risk of acute and cumulative toxicities may be increased. The main adverse effects of pemetrexed include myelosuppression, which may be prevented by folic acid and vitamin B12 supplementation. The most common non-hematologic side effect is elevation of hepatic enzyme levels. Other toxicities include rash, mucositis, nausea, and vomiting [2–5]. Pemetrexed nephrotoxicity is well known; however, its frequency is considered to be low [5]. The mechanism responsible for renal damage remains unknown. Recently, several cases of pemetrexed-induced tubular injury were reported in the international literature [6–10] including interstitial nephritis and fibrosis, as well as diabetes insipidus. Only a few patients were submitted to renal biopsy. We report a case of renal insufficiency as a leading cause of maintenance pemetrexed discontinuation for toxicity to advanced non-small cell lung cancer in routine practice.

## Case presentation

**Fig. 1:**
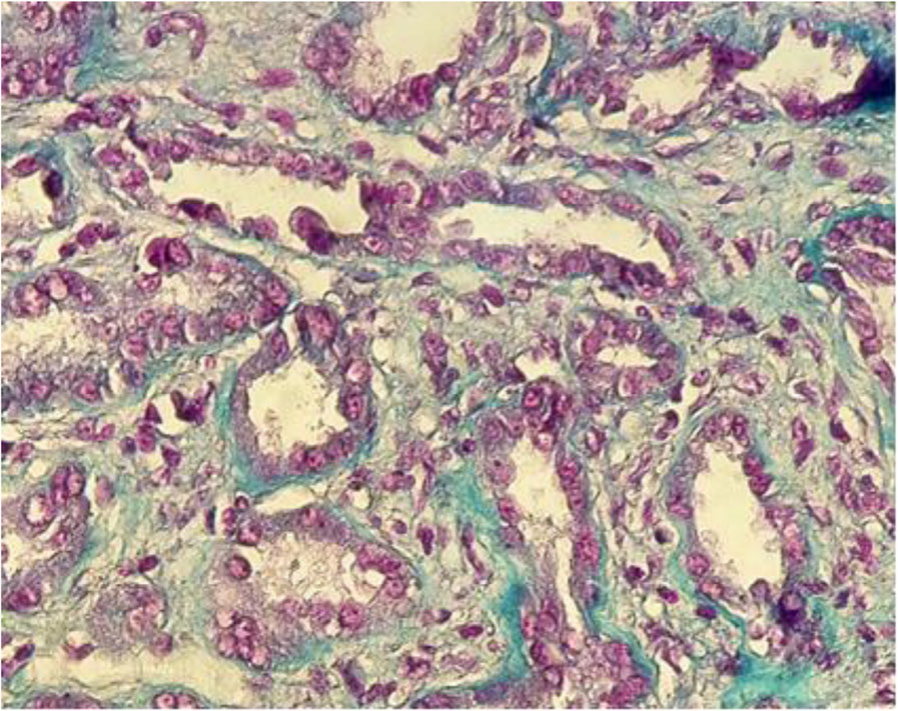
Kidney biopsy showed interstitial infiltration from many inflammatory cells, mainly mononuclear cells. (Hematoxylin and eosin stain, original magnification × 400. Masson’s trichrome)

A 69-year-old Moroccan man presented to our medical oncology department in January 2015. He was an ex-tobacco smoker (45 packs/year). His past medical history was unremarkable and he took no medications. His performance status was Eastern Cooperative Oncology Group (ECOG) 1. He was diagnosed as having primary advanced lung adenocarcinoma. Oncogenic driver mutations such as epidermal growth factor receptor or echinoderm microtubule-associated protein-like 4-anaplastic lymphoma kinase fusion gene were not performed. He was treated with first-line chemotherapy, including 3-hour paclitaxel infusions at dose (200 mg/m^2^, days 1) and carboplatin (area under the curve, 5), every 3 weeks for four cycles. After four consecutive 21-day cycles of chemotherapy, the disease had partially responded. He developed grade II peripheral neuropathy induced by paclitaxel according to Common Terminology Criteria for Adverse Events (CTCAE). He was then started on pemetrexed switch maintenance at dose of 500 mg/m^2^ (900 mg) every 3 weeks. He was premedicated with steroids and was receiving vitamin B12 injections. His serum creatinine (SCr) prior to pemetrexed administration was 0.8 mg/dl; Modification of Diet in Renal Disease (MDRD) estimated glomerular filtration rate (GFR) 85 ml/minute per 1.73 m^2^. After third cycle of pemetrexed infusion, his creatinine clearance (Cr Cl) started to drop, up to 25 ml/minute per 1.73 m^2^. Pemetrexed was stopped. Our patient was referred to a nephrologist. On admission, his blood pressure was 140/80 mmHg and a physical examination was normal. Sporadic consumption of non-steroidal anti-inflammatory drugs (NSAIDs) was reported. He was exposed to intravenously administered contrast from thoracoabdominal computed tomography (CT) in the week of his last pemetrexed treatment. Serum electrolyte levels and renal ultrasound were both normal. A 24-hour urine collection revealed a 0.7 g proteinuria without hematuria, glycosuria, leukocyturia, or proximal tubular dysfunction. Critical measures including maintaining adequate intravascular volume and mean arterial pressure, discontinuing pemetrexed drug, and eliminating exposure to nephrotoxins were performed. SCr level control was 2.2 mg/dl (MDRD estimated GFR 25 ml/minute per 1.73 m^2^). A kidney biopsy was performed. Renal biopsy specimen showed acute tubular necrosis with interstitial inflammatory infiltrate of mononuclear cells and interstitial fibrosis occupying 40% of renal cortex (Fig. 1). His SCr level was stable 4 months after the last dose of pemetrexed. He died 5 months later.

## Discussion

In patients with advanced lung adenocarcinoma, the results of phase III trials show that pemetrexed maintenance therapy allows longer survival [1, 2]. Given pemetrexed safety and simple intravenous administration every 3 weeks, this strategy seems to be of important clinical value. However, not all patients need or benefit from maintenance therapy. Events associated with decreased renal function were reported [3–5, 11]. In phase III trials, all grades of renal failure and grade 4 requiring dialysis were reported in 2.4 and 0.6% of patients, respectively [5]. Our case report describes renal insufficiency as a leading cause of maintenance pemetrexed discontinuation for toxicity to advanced non-small cell lung cancer in routine practice. Cr Cl decreased from 85 to 25 ml/minute per 1.73 m^2^ following the third pemetrexed infusion. This observed nephrotoxicity seems to be related to the mechanism of action of pemetrexed and its pharmacokinetics. Pemetrexed was eliminated as unchanged drug primarily from the kidney, by both tubular secretion and glomerular filtration with the former being the predominant mechanism for patients with normal renal function [12, 13]. Only a few patients were submitted to renal biopsy. Our patient experienced acute tubular necrosis and interstitial fibrosis following sequential treatment with pemetrexed. Our data are in agreement with other reports where renal injury has been reported after three, four, or six cycles of pemetrexed [6–10, 14, 15]. After discontinuation of pemetrexed, our patient’s renal function stabilized, but did not return to pre-treatment baseline. Although many acute kidney injury (AKI) risk factors were present (dehydration, NSAIDs, radiocontrast agents), none of them seemed to be responsible for kidney injury. The lapse of time between the administration of radiocontrast agents and AKI development excludes radiocontrast agent-induced nephropathy. Interstitial fibrosis was attributed to pemetrexed. However, we cannot exclude the contribution of these factors. Thus, pemetrexed must be considered an important cause of renal failure in patients with cancer. However, it is difficult to identify patients at higher risk of discontinuation based only on changes in laboratory values. Oncologists have to be vigilant in assessing their patient’s renal function for treatment.

## Conclusions

In conclusion, suggestions for mitigating the risk for renal toxicities during pemetrexed maintenance should be followed. Cr Cl must be measured before each cycle of pemetrexed administration. CT scans with contrast should be performed a few days to 1 week after pemetrexed administration. Patients must be appropriately hydrated during treatment. Concomitant medications should be reviewed with the patient and, when possible, medications that could potentially be nephrotoxic should be eliminated. A decision to use maintenance chemotherapy requires a discussion between patient and physician that adequately assesses the benefits of prolonged therapy and the impact in terms of toxicity and quality of life.

## Abbreviations

AKI: Acute kidney injury
Cr Cl: Creatinine clearance
CT: Computed tomography
CTCAE: Common Terminology Criteria for Adverse Events
ECOG: Eastern Cooperative Oncology Group
GFR: Glomerular filtration rate
MDRD: Modification of Diet in Renal Disease
NSAIDs: Non-steroidal anti-inflammatory drugs
SCr: Serum creatinine
